# Needle Aspiration and Cytology for Suspected Osteoarticular Tuberculosis in Children

**DOI:** 10.5704/MOJ.1811.007

**Published:** 2018-11

**Authors:** A Agarwal, S Singh, S Agarwal, S Gupta

**Affiliations:** Department of Orthopaedics, Super Specialty Paediatric Hospital & Post Graduate Teaching Institute, Noida, India; *Department of Pediatrics, Dr Ram Manohar Lohia Hospital and Post Graduate Institute of Medical Education and Research, New Delhi, India; **Department of Orthopaedics, Lady Hardinge Medical College, New Delhi, India

**Keywords:** osteoarticular tuberculosis, diagnosis, needle aspiration, cytology, microscopy

## Abstract

**Introduction:** Early diagnosis of osteoarticular tuberculosis (OATB) is essential to prevent significant functional disability. There is no single test for diagnosis. Despite an array of investigations available, definitive diagnosis at early stage before starting antitubercular drugs is still a challenge. **Materials and Methods:** A cross sectional study was carried out between February 2016 and October 2017. All children less than 18 years of age with suspected osteoarticular tuberculosis were included. The cases were subjected to simple needle aspiration from whichever site was accessible. Multiple sample aspirations were done at site of involvement. Smears were prepared from the aspirated material.

**Results:** Ziehl-Neelsen staining for Acid Fast Bacilli (AFB) showed deep pink red rods under light microscopy. Features suggestive of tuberculosis can be seen by May-Grünwald-Giemsa (MGG) staining. Auramine-O staining method of detecting AFB under fluorescent microscope shows the bacilli as greenish yellow slender curved rods in dark background. Fluorescent microscopy has higher sensitivity and comparable specificity. In our study, microbiological confirmation of OATB could be done in 100% cases where the lesion could be accessed for aspiration. The molecular techniques are relatively more expensive and not available everywhere.

**Conclusion:** Meticulous search for AFB in a well stained smear using three different staining methods provides a direct evidence of infection over costly imaging especially in poor patients seen in resource limited settings.

## Introduction

Global incidence of tuberculosis (TB) is approximately 9.6 million cases. Of these more than one third are in Asian countries: India, Indonesia, Myanmar, Thailand, Bangladesh, Pakistan, Sri Lanka, and Korea^1^. Extra-pulmonary TB (EPTB) accounts for approximately 17% of all TB cases and osteoarticular tuberculosis (OATB), makes up to 3% of all TB cases^2^. OATB is an aggressive disease and leads to joint destruction by spread of the infection from bone into adjacent joints and surrounding soft tissue. Early diagnosis is essential to prevent significant functional disability.

Despite an array of investigations available to a clinician, definitive diagnosis at early stage before starting antitubercular drugs is still a challenge in many cases, as presentation of OATB can mimic other diseases^3^. As per World Health Organization (WHO), every attempt should be made to confirm TB microbiologically^4^. Microscopic examination and staining of synovial fluid aspirate or aspirate from affected site is a simple and reliable investigation. Various staining methods have been used for demonstration of tubercle bacilli. Ziehl-Neelsen (ZN) staining for acid fast bacilli (AFB) shows deep pink red rods and beaded structure in oil immersion under light microscopy. May-Grünwald-Giemsa (MGG) staining may show features suggestive of TB: acellular eosinophilic granular material (caseous necrosis), Langhans type giant cells, mononuclear infiltrate (lymphocytes, plasma cells, macrophages). Fluorescent microscopy has higher sensitivity and comparable specificity which is further enhanced by concentration. Papanicolaou (PAP) stained smears under fluorescent microscope can also show AFB. Auramine-O staining under fluorescent microscope shows AFB as greenish yellow slender and slightly curved rods in dark background^5^. Now with the advent of newer inexpensive light emitting diode based fluorescent microscopes (LED-FM), which are easier to use, fluorescent microscopy can be widely used even in peripheral laboratories where culture facilities are not available^6^.

A study was conducted with the aim of arriving at an early and definitive diagnosis of suspected OATB in children with microscopy and staining without the need for costly imaging.

## Materials and Methods

A cross sectional study was carried out (with approval of the institutional ethics committee) during the period between February 2016 and October 2017. All children less than 18 years of age with suspected OA TB visiting the orthopaedic outpatient department were included in the study. Those patients who were clinically suspected of having acute pyogenic osteoarticular infection were excluded from the study. A preliminary workup was carried out in the form of complete blood count (CBC), erythrocyte sedimentation rate (ESR) and radiograph of the involved site. Wherever possible, the cases were subjected to aspiration with a 20 gauge or 22 gauge needle under all aseptic precautions after a written consent from the parents/guardians for the procedure. The procedure was done as a simple standard outpatient FNAC investigation. However, it was carried out by a cyto-pathologist in the presence of the treating orthopaedic surgeon to increase the accuracy of site. Multiple sample aspirations were done at the site of involvement as TB has patchy infliction and a representative sample may not be obtained in a single aspirate. Smears prepared from the aspirated material from affected site were air-dried for MGG stain and immediately fixed in methanol. After ZN staining, each slide was examined in oil immersion (1000x) under light microscopy for the presence of Mycobacterium tuberculosis (MTB) bacilli and other characteristic features of tuberculous infection. Additional smears were reserved for special staining procedures like Auramine-O staining and PAP staining method of detecting AFB under fluorescent microscope. The samples were analysed for CBNAAT wherever adequate sample was obtained (Table I). Advanced imaging like magnetic resonance imaging (MRI) was done only in cases where the patient could afford and if the site of involvement was inaccessible to simple outpatient fine needle aspiration cytology (FNAC).

**Table I:** Investigations done on aspirate material

Investigation	Frequency (n=7)
Light microscopy (MGG stain)	
Caseous Necrosis	5
Granuloma without necrosis	2
Giant cells	1
Lymphocytosis	5
Z-N stain	6 (86%)
Auramine O stain	7 (100%)
CBNAAT	3 (100%)

## Results

A presumptive diagnosis of OATB was made in 10 children with median age of 4.5 years (range: 1 year-17 years). Seven (70%) patients were females. The most common complaint was pain (60%), followed by pain and swelling in four (40%) patients at the affected site. Seven children had affection in easily accessible regions of the body. The remaining three cases were of spinal TB. ESR was elevated in all 10 patients (median: 52, range: 37-103). The demographic characteristics, laboratory and radiograph findings of these children are depicted in Table II.

**Table II:** Characteristics of children suspected with OA TB

Characteristics	Frequency (n=10)
Age in years (median, range)	4.5 (3-17)
Sex (male: female)	3:7
Presenting symptom	
Pain	6
Pain and swelling	4
Fever	2
Knuckle deformity	2
Site of lesion	
Clavicle	1
Ulna	1
Hip	1
Sacroiliac joint	1
Knee	1
Spine	3
Head of talus	2
ESR at 1st hour (median, range)	52 (37-103)
Radiograph	
Lytic area	2
Reduced disc space	1
Bony destruction and deformity	3
Sacroilitis	1
Normal	2
MRI findings s/o TB	4/4 (100%)

**Fig. 1:**
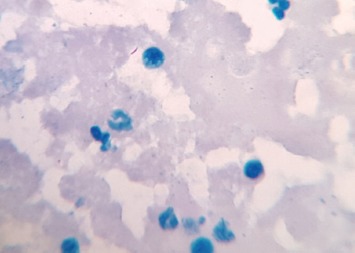
Mycobacteria in Ziehl Neelsen Stain (ZN stain, x1000).

**Fig. 2:**
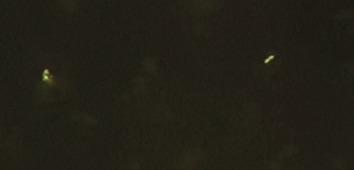
Mycobacteria in Auramine stain (Auramine stain, x400).

**Fig. 3:**
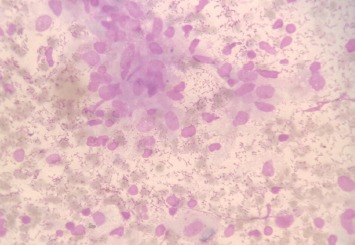
Epithelioid cell granuloma (MGG stain, x400).

Six of the seven samples obtained by needle aspiration were positive for AFB (ZN stain) (Fig. 1). The remaining one was doubtful but it showed AFB with Auramine-O staining. In one particular case, there was a single TB bacillus on ZN staining where Auramine-O also showed positive result. All seven cases were positive for MTB bacteria with Auramine-O stain (Fig. 2). MGG staining (Fig. 3) was done in all cases. CBNAAT could be done in only three of the seven patients where adequate sample was obtained. All were positive for MTB and sensitive to rifampicin.

**Fig. 4:**
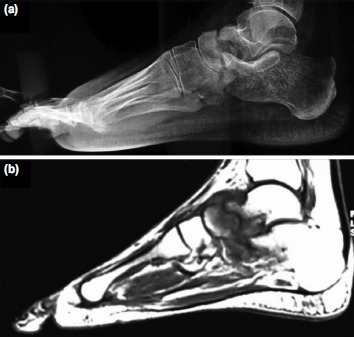
(a) Radiograph showing bony destruction of head of talus. (b) MRI (T1W) of same patient. and even air-drying artifacts in Papanicolaou stained smears. Also, there is a need for slides to be screened within a short period of preparation as the fluorescence diminishes over time. In addition, Auramine-O permanently destroys cell morphology making further morphological review impossible^22^. Thus, fluorescence microscopy is an important adjunct to visualisation using ZN stain on light microscopy and not a replacement.

Out of the ten cases, MRI was advised or had been carried out in only three cases. One patient had sought consultation with MRI already done elsewhere. MRI showed features suggestive of TB (cortical erosion, signal enhancement in T2W vertebral images, associated soft tissue thickening, perifocal soft tissue oedema, and pre- and/or paraosseous collection with granulation tissue) in all cases (Fig. 4b).

In the current study, four patients have completed anti-tuberculous treatment (ATT) of 15 months and are asymptomatic. Remaining six patients are in different phases of anti-tubercular therapy showing signs of recovery, with relief from constitutional symptoms and no further complications. Adverse effects to ATT was not noted in any of the patients.

## Discussion

There is no single test to prove the diagnosis of OATB Hematological parameters like elevated erythrocyte sedimentation rate (ESR) and lymphocytosis may aid in the diagnosis but are neither specific nor reliable. A positive Mantoux test favors the diagnosis but again is non specific^7^. Radiographs give a good clue to diagnosis, but sufficient bony destruction must have occurred to be visible on the radiograph (Fig. 4a). MRI when available is a valuable adjunct to plain radiographs. In conjunction with a high index of suspicion, in an endemic country like India, MRI diagnosis of "most likely tuberculous in origin", can often be made safely as certain characteristics are unique^8^. However, MRI cannot differentiate between pyogenic and tuberculous infections. In most cases, when the disease is in the early stages, MRI diagnosis too, cannot be entirely relied upon^9^. MRI is costly and difficult to advocate in poor patients or where facilities are scant. Microscopy is still the most widely used tool for TB screening.

In our study microbiological confirmation of OATB could be done in 100% cases where the lesion could be accessed for needle aspiration. As per WHO guidelines, diagnosis of EPTB should be based on at least one specimen with confirmed MTB or histological or strong clinical evidence consistent with active EPTB^4^. Organism isolation is not only important for definitive diagnosis but also for determining phenotypic drug susceptibility testing (DST) by culture. Although culture is the gold standard for diagnosis of TB, the culture positivity rate in case of OATB is very low (10-20%)^10, 11^. Moreover, it is difficult to obtain adequate sample for culture with needle aspiration, as in our study. Also, the sensitivity of MTB detection by culture isolation for children thought to have clinical disease is much lower than that for adults due to the paucibacillary nature of paediatric disease. The limited sensitivity of culture as well as the rapid progression to disease in children necessitates that the decision to initiate treatment for TB be made as early as possible prior to microbiological culture confirmation which requires long periods (two to four weeks on average)^12^. Although new techniques are available to optimise the culture system which dramatically shorten this delay (72 hours), but this requires costly dedicated controlled setups not available everywhere^13, 14^.

Direct staining for AFB has been reported as the most rapid diagnostic method^15^ but, the accuracy of microscopic examination largely depends on the specimen containing a sufficient number of bacteria (>104/ml). Various studies have been conducted for diagnosis of OATB by demonstration of tubercle bacilli (Table III). Needle aspiration is a sensitive technique and has high positivity rates for detection of AFB as observed in our study as well as by Arathi *et al* (75%) and Masood *et al* (63%)^16, 17^. However, Mousa *et al* found a low rate (27%) of positivity for AFB in their study. This might possibly be because many samples were obtained from sinus tract rather than the site itself and subjected to Gram and ZN staining^18^. Needle aspiration should be done with onsite guidance of the treating orthopaedician to obtain adequate and representative sample. Moreover, multiple aspirates increase the adequacy and accuracy of the sample.

**Table III:** Comparison of different studies for diagnosis of Osteoarticular TB by demonstration of Tubercle Bacilli-

S. No	Researcher	No. of cases	Site	Method	Method of demonstration	Positivity of TB	Study design; Age group (range in years)
1	Masood *et al*^20^ (1992)	11	Lesion	FNAC	Giemsa staining	73%	Observational; Adult patients (21-65 years)
					ZN Staining	64%	
					Culture	83%	
2	Mousa *et al*^21^ (1998)	22	Sinus Tract	Aspiration	ZN stain (6)	27.3%	Observational; Adult patients (26-82 years)
					Culture (14)	63.6%	
					Histology (5)	22.7%	
3	Enache *et al*^23^ (2005)	20	Lesion	Surgical Biopsy Specimen	Classical staining technique with haematoxylin- eosin (18)	90%	Retrospective; All age groups (1-69 years)
					Immuo histochemistry (2)	10%	
4	Singh *et al*^24^ (2006)	12	Lesion	FNAC	Giemsa staining	75%	Observational; All age groups (5-30 years)
					ZN staining	100%	
5	Muangchan and Nilganuwong *et al*^13^ (2009)	99	Lesion	Biopsy	Histopathology	46.5%	Retrospective; All age groups (1-85 years)
					ZN staining	40.4%	
					PCR	33.3%	
					Culture	19.2%	
6	Arathi *et al*^19^ (2013)	16	Lesion	Surgical Biopsy Specimen	H&E stain (16)	100%	Retrospective; All age groups (6-60 years)
					ZN stain (4)	75%	
7	Our Study (2018)	10	Lesion	FNAC	MGG stain (5/7)	71%	Observational; Paediatric (1-17 years)
					ZN stain (6/7)	86%	
					Auramine stain (7/7)	100%	

Conventionally, ZN staining has been widely used for staining AFB. Simple microscopy cannot distinguish MTB from atypical mycobacteria^15^. On the contrary, fluorescence microscopy with fluorochrome dyes such as Auramine-O or Auramine–rhodamine is known to possess higher degrees of sensitivity and specificity and hence this method is considered as more accurate test for the diagnosis of TB. Even with small sample (as low as 20 μL) and bacilli concentration <106/ml, TB bacilli can be detected without loss of sensitivity. Treatment with heat or decontaminating chemical agents do not interfere with the autofluorescence. Moreover, it is rapid and easy to perform even in less trained hands^19^. Auramine-O stain is now being adapted as the preferred method for centres with high workload as it has the advantage of being less laborious. Bacteria fluoresce in dark background and are easier to count^20^. Fluorescence microscopy has minimal power requirements and has ability to run on batteries. Additional advantages of this technology are that lower magnifications can be used, enabling rapid screening over a wider area of the smear to be seen and resulting in up to four times faster examination and lower maintenance requirements. Thus, this technology is believed to be more beneficial in high-burden resource-limited settings. As a result, WHO recommended the introduction of LED-FM in both high and low-volume laboratories^21^. However, Selepe *et al* reported problems in the detection of MTB using fluorescent methods which include fluorescence that can be displayed by some non- mycobacterial microorganisms such as *Bacillus subtilis Staphylococcus aureus*, Nocardia, yeasts, the presence of small naturally fluorescent particles in smears or in poor quality coverslips

In our study, light microscopy findings suggestive of TB like epithelioid granulomas were seen in two cases (25%) and caseous necrosis in five cases (75%). Enache *et al*, in their 19-case series document biopsy smears diagnostic of OATB in 18 cases which showed epithelioid granulomas and caseous necrosis^23^. Singh *et al* detected granulomas with or without necrosis in 75% cases and demonstrated AFB on ZN staining in 100% cases. They stressed on thorough search for MTB in stained slides^24^. In the series by Muangchan and Nilganuwong diagnosis by biopsy was done in 46.5% of cases^10^.

The enzyme-linked immunosorbent assay (ELISA) has a reported sensitivity of 32% with EPTB against the mycobacterial antigen A60^25^. The technique of DNA amplification (CBNAAT/Genexpert) by polymerase chain reaction (PCR) has further improved sensitivity by detecting the presence of extremely small quantities of MTB in clinical samples^26^. Genexpert has overall sensitivity of 95.7% and specificity of 99.3% and potential of drug sensitivity testing (DST) with reference to Rifampicin to the tune of 99%^27, 28^. It is however, not widely available, is costly, has limitation of requirement of stable electrical power supply, temperature control, annual calibration of instrument and requirement of at least 1 ml of sample^29^. Metaferia *et al* compared sensitivity of detection of EPTB on LED-FM smear examination versus genexpert^30^. They found that 71% of smear negative cases were genexpert positive, which they ascribed to use of unprocessed sample for smear examination. There was no false positive case on smear examination. Hence, they opined that genexpert should be considered as an adjunct test for improved case detection in smear-negative EPTB suspects in low income settings, instead of replacing smear microscopy.

In cases with high degree of clinico-haematological suspicion for OATB, taking into account the simplicity of the needle aspiration and cytology and rapidity of diagnosis, it can replace costly imaging modality as an alternative procedure before starting ATT. Meticulous search for AFB must be done on a well stained smear. Though an old technique, it proves to be of great value in diagnosing TB in developing countries of south and south-east Asia.

Since we performed the study only on paediatric OATB cases, the sample size in our study was relatively small, which is a limitation. Further studies need to be conducted before we can statistically claim 100% detection rate with our method. Nevertheless, in the background of studies done previously (Table III), it can be said that using the three staining methods, the accuracy and reliability of detecting MTB by simple smear microscopy increases. If the aspirate sample taken for smear is enough, it can be subject to genexpert and culture which increase the reliability of diagnosis and also gives additional information about drug sensitivity testing.

## Conclusion

Early diagnosis and treatment of OATB in children is essential to prevent joint deformities. Cytopathological examination of multiple aspirates of affected region is highly sensitive when performed in conjunction with the pathologist. Meticulous search for AFB with different staining methods provides a direct evidence of infection over costly imaging especially in poor patients seen in resource limited settings.

## Conflict of Interest

The authors declare no conflicts of interest.
